# Structure- and Solvent-Property Relationships for the Electronic Energies of Charge-Transfer Complexes Between Certain Benzene Derivatives*

**DOI:** 10.6028/jres.080A.019

**Published:** 1976-04-01

**Authors:** Harold Argentar

**Affiliations:** Institute for Materials Research, National Bureau of Standards, Washington, D.C. 20234

**Keywords:** Aromatic amine, benzene derivative, charge-transfer complex, dental materials, electron affinity, electron spin resonance spectroscopy, extrathermodynamic relationships, ionization potential, linear free-energy relationship, polarography, solvatochromic relationship, ultraviolet and visible spectroscopy

## Abstract

A chemical model is proposed for describing charge-transfer complexes between aromatic amines and electron-accepting benzene derivatives containing a group having a double- or triple-bond conjugated with the benzene ring. According to this model, an electron migrates from the nitrogen atom of the amine to one of the atoms of the multiple-bonded group during charge-transfer interaction.

Structure-property relationships were derived for correlating: (1), the transition energies of the complexes; (2), the ionization, or oxidation, potentials of the amines, and (3), the electron affinities or reduction potentials of the electron acceptors, with the electron-donating abilities of the substituents of the various compounds. Transition energies calculated from reported spectroscopic data for these complexes were correlated using equations derived in this study. Similarly correlated were reported data for the above properties of the amine and electron acceptor.

Equations were derived for correlating the effect of variation in solvent on the transition energies of the complexes. Correlation of reported spectroscopic data indicated that the greatest effect is caused by variation in the refractive index; of secondary importance was the change in dielectric constant.

## 1. Introduction

**Figure 1:**
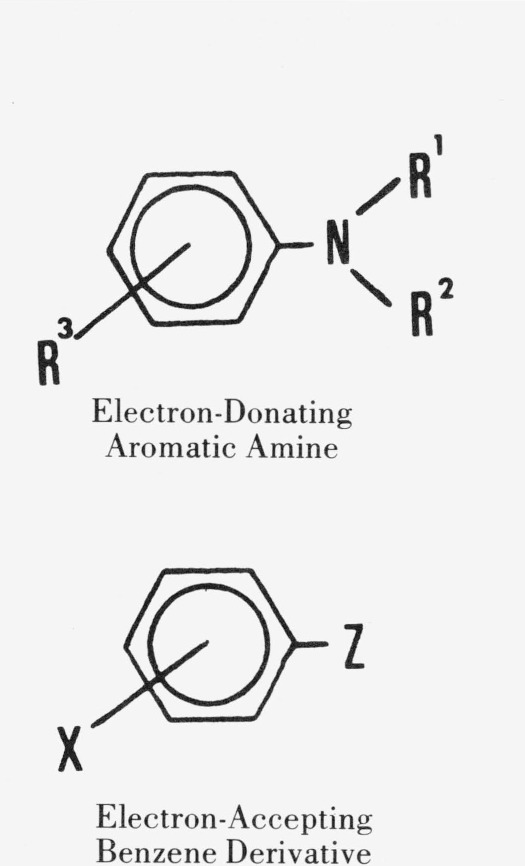
Components of the Electron Donor-Acceptor Complex Studied in This Investigation. *R*^1^ and *R*^2^ are either H or *CR*′*R*″*R*′″, where *R*′, *R*″ or *R*′″ is any atom or group of atoms that is sufficiently small to allow for coplanarity of the benzene ring and the nitrogen substituent. *R*^3^ is any substituent in the *meta*- or *para*-position; if in the *ortho*-position, the substituent must be small enough to allow for coplanarity of the benzene ring and the nitrogen substituents. Z is a group of atoms containing a double- or triple-bond conjugated with the benzene ring. X is any substituent, and X may be repeated so that multiply-substituted compounds are considered.

A number of reports on investigations of electron donor-acceptor (EDA) complexes in which aromatic amines function as the electron donor and other benzene derivatives function as the electron acceptor have appeared in the last twenty years [1–24].^1^ (In this investigation, the term “aromatic amine” is limited to aniline and its derivatives, while compounds like benzylamine and naphthylamine are excluded.) Figure 1 shows the structures of the components of the complex discussed.

Practical application of the complexes between aromatic amines and electron-accepting benzene derivatives have been quite diverse in materials science and applied chemistry. In one investigation, the complexes were prepared for the purpose of obtaining materials with a greater mechanical strength than that possessed by the isolated components of the complex [17, 18, 21]. In other cases, the complexes were used as a tool for studying certain biochemical processes and in an analytical procedure [11, 13, 23, 24]. In some instances, complexes between aniline derivatives and other aromatic compounds have been shown to have undesirable properties, e.g., a considerable amount of color when present in otherwise acceptable composite filling materials designed for use in dentistry [25, 26].

Although such donor-acceptor complexes are of fundamental interest and have practical utility, an overall view of the physiochemical properties of these complexes as they vary with the natures of the substituents of the electron-donating amine and the electron acceptor and of the solvent has apparently not heretofore been undertaken. If a quantitative relationship between a measured physical property and structural and solvent parameters were effected, then a complex could be designed having the value of the physical property optimized over all possible variations in structure.

One of the most important properties of these complexes is the appearance of an intermolecular electronic charge-transfer (*c-t*) transition observable by ultraviolet or visible light spectroscopy [1, 4]. The term “chargetransfer complexes” has therefore been applied to these complexes as well as to others that exhibit this phenomenon [27]. “The property of charge-transfer complexes which is normally most readily, and certainly most frequently, measured is the energy of the (usually lowest) intermolecular charge-transfer transition of the complex in solution” [28]. This energy, *E_ct_* is readily obtained by spectroscopic measurements using the relationship [29]:

$$E_{ct}=N_{0}hv_{\max}^{ct}=N_{0}hc/\lambda_{\max}^{ct},$$ (1)

where *E_ct_* is the *c-t* transition energy per mole of the complex, *N*_0_ is Avogadro’s number, *h* is Planck’s constant, $v_{\max}^{ct}$ is the frequency at which the differential absorption of the complex is a maximum [5], *c* is the speed of light in a vacuum and $\lambda_{\max}^{ct}$, the wavelength corresponding to $v_{\max}^{ct}$. The differential absorption is obtained by measuring the absorption of the complex relative to the sum of the absorptions of the individual components rather than to the absorption of the solvent as in the usual case [26].

It must be clearly realized that in addition to the charge-transfer transition occurring in a thermodynamically stable donor-acceptor complex, charge-transfer transitions may occur during a random encounter of the donor and acceptor. Orgel and Mulliken have referred to this phenomenon as “contact chargetransfer” [30]. If only contact charge-transfer occurs in a given material, then the experimentally determined thermodynamic formation constant for the hypothetical complex assumed to be present would be found to be essentially zero. For economy of words, both stable complexes and those interactions demonstrating charge-transfer transitions will be referred to as “charge-transfer complexes”, or simply, “complexes”. The results obtained in this investigation are thus not necessarily related to the thermodynamics of EDA complex formation and should be kept separate.

The principal purposes of this investigation were: (1), to derive equations suitable for correlating the electronic transition energies of complexes (in solution) between aromatic amines and compounds from one class of electron-accepting benzene derivatives with the properties of the substituents of the two compounds and the nature of the solvent and (2), to use these relationships for correlating previously reported transition energies (or transition energies calculated from previously reported frequencies, or wavelengths, at which maximum spectroscopic absorption occurs) of the complexes. The electron acceptors considered here are exemplified by aryl-substituted nitrobenzenes, cyanobenzenes, and benzoates, i.e., compounds in which an electron-accepting group possesses a double- or triple-bond that is conjugated with the benzene ring. This class contains those benzene derivatives that most readily accept electrons, e.g., 1,3,5-trinitrobenzene and 1,2,4,5-tetracyanobenzene.

In the course of this investigation, comparable equations were derived for correlating the ionization potentials, or oxidation potentials, of the aromatic amines and the electron affinities, or reduction potentials, of the aromatic compounds possessing a common electron-accepting group with structures in the respective series of compounds.

## 2. Transition Energies and the Natures of the Electron Donor and Acceptor — An Overview

Several equations have been derived relating the *c-t* transition energy to the natures of the donor and acceptor [31 and references contained therein]. The simplest equation and probably the first to be derived is:

$$E_{ct}=N_{0}\left(IP-EA+C\right),$$ (2)

where *IP* is the ionization potential of the electron donor, *EA* is the electron affinity of the acceptor and *C* is a collective term for the solvation, polarization, and nonbonding contributions to the energy [32, 33]. As originally conceived, *N*_0_ in eq (2) is absent and thus the energy computed is for one molecule of complex. As will be seen shortly, use of *N*_0_ provides for the calculation of the energy on a molar basis, which is more convenient for correlation of chemical data.

## 3. Identification of the Reaction Sites of the Electron Donor and Acceptor

It will become apparent from the next section that, in order for the transition energies to be correlatable by the usual structure-property relationships, it is necessary to identify the reaction sites in the electron donor and acceptor and to determine if they remain the same as the structures of the reactant molecules change.

The first (and most generally accepted) hypotheses to be published regarding the locations of reaction sites in various molecules forming charge-transfer complexes were those by Mulliken in 1952 [34]. He considered electron transfer from an aliphatic amine to involve the non-bonded electrons on the nitrogen atom and therefore designated this class of compounds as *n*-donors. Similarly, if electron transfer to a vacant orbital of an electron acceptor occurred, then the acceptor was labeled a *υ*-acceptor. However, on the basis of theoretical considerations, he concluded that *all* aromatic compounds donate or accept an electron during chargetransfer complexation primarily, if not solely, at the *π*-electron system of the benzene ring. That is to say, there is no one single reaction site although, of course, one location may be somewhat more reactive than another. He thus designated these compounds as *π*-donors or *π*-acceptors.

This assumption with respect to the nature of aromatic electron acceptors has been used in several studies on charge-transfer complexes [26, 33]. However, Tsubomara concluded that in charge-transfer complexes of aniline derivatives with an iodine molecule that the amine can function as an *n*-donor [35].

In order to reach a definite conclusion as to whether the aromatic amines and acceptors in this study involve the *π*-system or *n*-electrons or *υ*-orbitals, direct experimental evidence is required. Unfortunately, this is not available. However, indirect evidence has been obtained that can be used to provide a reasonable assumption concerning the locations of the reaction sites. Recently, Janzen reviewed the literature on the electron spin resonance spectra of free radicals formed from benzene derivatives on gaining or losing an electron [36]. He indicated that an aromatic amine cation radical has the odd electron primarily located on the nitrogen atom. Likewise, an anion radical formed from a substituted nitrobenzene, cyanobenzene, or other benzene derivative possessing a group having one double- or triple-bond conjugated with the benzene ring generally has the odd electron on the atom on the group adjacent to the benzene ring, i.e., *α*-atom, although in some cases, the odd electron may reside on the *β*-atom.

Another approach for obtaining the location of the odd electron in aromatic radical anions has been reviewed by Hayon and Simic [37]. In water, anion radicals produced from the reaction of electrons (generated in pulse radiolysis of the solvent) with various compounds are basic and can protonate. Protonation of the anion radicals derived from benzoic and terephthalic acids and those from benzoyl esters occurs at the carboxyl group and not in the aromatic ring. Consistent with this is that protonation of anion radicals formed from nitrobenzene and aryl substituted nitrobenzenes takes place at the nitro group. In addition, the protonated anion radicals of benzaldehyde, acetophenone and benzophenone produced indirectly exist as *α*-hydroxyalkyl radicals. Consequently, in anion radicals of these highly electrophilic compounds, the odd electron is localized on the substituent and not the ring.

Meisel and Nata [38] have very recently stated that “electron transfer to a nitro compound and from its radical anion is expected to involve the nitro group as the main site of the transfer.” Their experimental results were consistent with this assumption.

**Figure 2:**
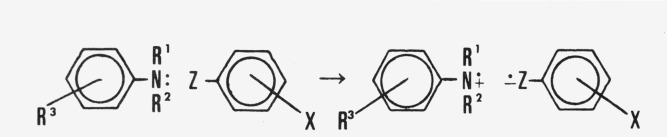
The formation and structure of the aromatic amine charge-transfer complex of benzene derivatives containing an electron-accepting group. Not depicted are the canonical structures showing the resonance stabilizing of the electronic charge by the aromatic rings.

Based on all these considerations, it will be assumed that the charge-transfer complex between an aromatic amine and a benzene derivative containing a group having a double- or triple-bond conjugated with the ring is as shown in figure 2. That is to say, electron transfer occurs between the nitrogen atom of the aniline derivative and one of the atoms of the group containing the multiple-bond, i.e., *n-v* complexation, in Mulliken’s terminology.

## 4. Transition Energies and Extrathermodynamic Relationships

In order to correlate the thermodynamic properties (e.g., equilibrium constants and enthalpy) or the kinetic data (e.g., rate constants and activation energies) with chemical structure for reactions of a series of closely related compounds in which the reaction site is held constant and various nonreacting atoms or groups of atoms are substituted in the molecule, chemists have utilized the concept of the “extrathermodynamic relationship” [39, 40 and references therein]. (Since one major use of this concept is in correlating the free energy changes in a group of compounds during reaction, the term “linear free-energy relationship” has also been applied but it is probably best to use this terminology in its more restrictive sense.)

According to this treatment of the data, each substituent has a numerical value, i.e., sigma value, that reflects the ability of the substituent to donate electrons to the reaction site and which is independent of the reaction conditions, e.g., temperature and solvent. The sigma value will, however, depend on: (a) in aromatic compounds, the position of the substituent in relationship to the reacting site, i.e., *ortho*, *meta*, or *para* (abbreviated as *o*, *m*, *p*, respectively); (b) the nature of the intervening atoms, i.e., aliphatic versus aromatic molecule; and (c) in some rather unusual circumstances in aromatic molecules, if direct conjugation with the reacting site occurs.

In the usual case, i.e., the reaction of *meta*- and *para*-substituted benzoic acid, the relationship is generally given as

$$\log\;K/K^{0}=\rho \sigma ,$$ (3)

where *K* is the reaction rate, or equilibrium constant; *K*^0^ is the corresponding value for the compound in the series chosen as the standard; *ρ* is a parameter that depends on the reaction and the reaction conditions, and reflects the relative sensitivity of the reaction rate or equilibrium constant to variations in the substituent constant *σ* (“sigma”) previously described. The standard compound is benzoic acid and *σ* for the aromatic hydrogen atoms is defined as zero.

For *ortho*-substituted derivatives, two effects come into play: (1) the ability of the substituent to donate electrons, and (2) the steric interaction of the substituent on the reaction site [41]. This site generally is a group of atoms that requires a specific geometrical relationship with respect to the plane of the aromatic ring. If the substituent is small or is sufficiently flexible that no steric hindrance results, then *σ_o_–σ_p_* is approximately constant, as in one series of benzoic acid derivatives [41]. If the substituent is so large that it interferes with the reacting site then no simple relationship between these *σ* values is possible. This latter case is beyond the scope of this investigation.

For reaction of aromatic compounds, e.g., phenols and anilines, in which a negative charge that is in direct conjugation with the substituent in the *para*-position is created or destroyed, the appropriate equation is:

$$\log\;K/K^{0}=\rho \sigma^{-}.$$ (4)

(For most substituents, the values of *σ* and *σ*^−^ are equal.)

For reactions of aromatic compounds, e.g. benzylic carbonium ions, in which a positive charge that is in direct conjugation with the *meta*- or *para*-substituent is generated or eliminated, the appropriate equation is:

$$\log\;K/K^{0}=\rho \sigma^{+}.$$ (5)

For reactions in the aliphatic series, in which the substituent is bridged by methylene groups to the reacting site, the appropriate equation is:

$$\log\;K/K^{0}=\rho \sigma^{*}.$$ (6)

Tables of the values of the substituent constants have been compiled and are readily available [41, 42].

A heuristic approach will be used to derive the corresponding relationships involving charge-transfer energies. It has previously been shown for a series of methine dyestuffs formed from an organic acid that the low level transition energies (*λ*_max_ ~ 450–600 nm), which have been assigned to an intramolecular *c-t* process, of the dyes vary with the logarithms of the ionization constants, *K_A_*, of the parent acids by the relationship [43, 44]:

$$N_{0}hv_{\max}-2.303\;RT\;\log\;K_{A}=\text{constant},$$ (7)

where *v_max_* is the frequency at which the spectroscopic absorption of the dye is maximum, *R* is the gas constant and *T* is the absolute temperature. Solving eq (7) for log *K_A_* gives:

$$\log\;K_{A}=\frac{N_{0}hv_{\max}}{2.303\;RT}-\frac{\text{const}}{2.303\;RT}.$$ (8)

Assuming that *K_A_* can be correlated with the electron- donating ability of a substituent by one of the previous equations, eqs (3)–(6), gives:

$$\frac{N_{0}hv_{\max}}{2.303\;RT}-\frac{N_{0}hv_{\max}^{0}}{2.303\;RT}=\rho \sigma^{x},$$ (9)

where $v_{\max}^{0}$ corresponds to $K_{A}^{0}$ and *σ^x^* represents *σ*, *σ*^−^ etc. as the case may be.

In the charge-transfer complexes considered in this investigation, it was hypothesized that electron loss occurs at the nitrogen atom of the aromatic amine and electron acceptance occurs at the multiple-bond conjugated with the benzene ring of the second compound (see previous section). For such complexes between one series of aromatic compounds in which only one type of substituent is varied, e.g., the nitrogen substituent of the amine or the aryl substituent a nitrobenzene, and the second aromatic compound kept constant, it is reasonable to assume that an equation similar in form to Equation (9) is applicable. This is so because in both cases, the reaction sites are fixed and in varying the substituents, families of compounds are obtained. Therefore, the appropriate equation for correlating the *c-t* transition energies of the EDA complexes in this investigation with substituent electron-donating abilities is obtained by replacing *v*_max_ with $v_{\max}^{ct}$ giving

$$\frac{N_{0}hv_{\max}^{ct}}{2.303\;RT}-\frac{N_{0}h{\left(v_{\max}^{ct}\right)}^{0}}{2.303\;RT}=\rho \sigma^{x}.$$ (9′)

In the only investigation in which a comparable equation was applied, the *c-t* transition energies of 1-alkylpyridinium iodides with various substitutents in the 3- and 4-positions of the pyridinium ring were correlated by assuming that the 3- and 4-positions were comparable to the *meta*- and *para*-positions, respectively, of a benzene derivative [45–47]. Although used in the form:

$$\frac{E_{T}X-E_{T}}{2.303\;RT}=\rho \sigma^{-},$$ (9′A)

where *E_T_X* is the transition energy of the substituted compound and *E_T_*, that of the parent, it is written in the abstract of ref. [46] and in ref. [47] as:

$$\frac{E_{T}}{2.303\;RT}=\rho \sigma .$$ (9′B)

(When the *σ* and *σ*^−^ values differed, Kosower used the *σ*^−^ values and referred to them as “resonance” *σ* values.) A value of – 13.4 was reported for p [45–47].

Because the term for the equilibrium free-energy change or its analog in kinetic or spectroscopic data can be put into the form Δ*G*/2.303*RT* where Δ*G* represents the free-energy change or the kinetic or spectroscopic counterpart thereof, in extrathermodynamic relationships [39, 40], it will be useful to denote this function of *G* simply as *pG.* This expression will be called “the extrathermodynamic function” of *G.* This convention follows the concept discussed by Sillen [48] in that instead of the electrical potential *e* of a half-cell, sometimes the dimensionless quantity *pE* is used, which is defined by:

$$pE=\frac{eF}{RT\;\ln\;10}=\frac{eF}{2.303\;RT’}$$ (10)

where *F* is Faraday’s constant.

Thus eq (9′) can be written as:

$$pE_{T}-pE_{T}^{0}=\rho \sigma^{x}.$$ (9′C)

This function of the transition energy as well as others to be discussed for extrathermodynamic relationships is given in table 1.

It has been shown previously that if the extrathermodynamic relationship is fitted in the forms indicated in eqs (3)–(9′C), infinite statistical weight is placed on the measured value of the parent compound [49, 50]. To avoid this, it was proposed that the regression line fitting the data not be forced to go through the origin, i.e., that there be an intercept calculated from the data [49, 50]. Therefore, the extrathermodynamic relationships should (and will in this investigation) be expressed in the general form

$$pE_{T}=pE_{T}^{0}+\rho \sigma^{x},$$ (9′D)

where both $pE_{T}^{0}$ and *ρ* are obtained by least-squares fitting the equation.

## 5. Ionization Potential of Amines and Extrathermodynamic Relationships

The effect of the amine structure on the *c-t* transition energy of the complex is probably due primarily to variations in the ionization potential of the amine donor as indicated in eq. (2). Therefore, the correlation of the ionization potential of the amine with structure is an important area of inquiry.

### Effect of Varying the Nitrogen Substituents

For aromatic amines, two separate structural changes are possible: nitrogen substitution and ring (aryl) substitution. The effect of substituents on the ionization potentials of aliphatic amines is highly correlated with that of alkyl free radicals [51]. Thus a straight line is obtained if the ionization potentials of aliphatic amines with the formula *R*^1^*R*^2^*R*^3^*N* is plotted against those of the corresponding methyl free radical, i.e., *R*^1^*R*^2^*R*^3^*C* · [51]. In addition the ionization potentials of these latter free radicals are approximately linearly related to the sum of the σ* constants, ∑σ*, of the substituents on the carbon atom possessing the odd electron [51]. However, Poldoja [52] proposed that for such systems hyperconjugation effects must be considered and proposed the equation:

$$IP=H\left(n\right)+\alpha n_{1}+a\Sigma \sigma *,$$ (11)

where *H(n*) are the intercepts, which may vary with the number of protons on the atom possessing the odd electron; *n*_1_ is the number of protons on the carbon atom of the alkyl substituent next to the atom with the odd electron, and *α* and *a* are parameters obtained by least-squares fitting the equation.

To obtain the corresponding extrathermodynamic relationship, eq (11) is substituted into eq (2) and *C* is assumed to be linearly related to the ionization potential, giving:

$$E_{ct}=N_{0}\left[H^{\prime }\left(n\right)+\alpha^{\prime }n_{1}+a^{\prime }\Sigma \sigma *-EA\right].$$ (12)

Therefore:

$$pE_{T}=\frac{E_{ct}}{2.303\;RT}=H^{\prime \prime }\left(n\right)+\alpha^{\prime \prime }n_{1}+\rho \Sigma \sigma *,$$ (12′)

where the value of *EA* is contained in the intercepts *H*″(*n*).

The data of Foster and Hammick [1] for complexes of *N*-substituted anilines with 1,3,5-trinitrobenzene in cyclohexane were fitted by eq (12′) (the contribution of the aromatic portion of the amine to ∑σ* was ignored since it was constant throughout this series) using indicator variables [53] to obtain the intercepts *H*″ (*n*) and the results are shown in table 3. (In what follows, whenever no reference is indicated after it is mentioned that data were correlated, the correlation was performed by the author. The results in the tables were obtained by the author). Since *H*″ (*n*) are not significantly different for the various amines (95% confidence level) [53] and *α*″ is negligible, the regression was repeated using the relationship:

$$pE_{T}=pE_{T}^{0}+\rho \Sigma \sigma *.$$ (13)

The results are shown in table 3. An analysis of variance [54] infers (at the 95 percent confidence level) that the goodness-of-fit eq (13) is consistent with that of eq (12′) and, therefore, eq (13) describes the data adequately.

An implicit assumption made in the derivation and use of eq (13) is that the substituent produces no steric interference with the benzene ring. Just as *ortho*- substituents that are bulky can cause serious problems in correlating thermodynamic data (sec. 4), some nitrogen substituents may also prove to be anomalous. Hickinbottom [55] found that tertiary aromatic amines such as *N*-methyl-*N-t*-butylaniline and *N*-methyl-*N*-*t*-amylaniline are as unreactive with nitrous acid as is *N,N*-dimethyl-*o*-toluidine, which possesses a bulky methyl substituent in the *ortho*-position, although other tertiary aromatic amines possessing an unsubstituted *para*-position are generally quite reactive in forming the *p*-nitroso derivative [56]. Primary alkyl substituents such as CH_2_R, where R may be any atom or group of atoms, attached to the nitrogen atom produce no apparent steric problems in aniline derivatives with no *ortho*-substituents. If the alkyl substituent is secondary, it is not known if steric interference with the ring occurs. A further discussion may be found under “*Ortho Substitution*” below.

### Effect of Varying the Ring (Aryl) Substituents

Although few investigations on the effect of varying the nitrogen substituent have been made, a number of them have indicated the effect of ring substitution. Since the ionization potentials of alkyl free radicals are linearly related to the ionization potential of the corresponding substituted aliphatic amines [51], it would not be surprising to find that the ionization potentials of aryl-substituted benzylic free radicals are linearly related to those of the correspondingly ring-substituted anilines possessing nitrogen substituents held constant throughout the series under comparison. However, the correspondence does not appear to have been made previously.

In the case of *meta*- and *para*-substituted benzylic free radicals, the ionization potential is linearly related to *σ*^+^ of the ring substituent [57]. Crable and Kearns [58] found that for *para*-substituted anilines, the ionization potential is linearly related to *σ^+^.* A plot of their data (not shown) for *meta*- and *para*-substituted anilines shows that by excluding the *meta*- and *para*-amino substituted compounds, the correlation between ionization potential and *σ*^+^ is quite good. This plot indicates that the maximum deviation (if the amino substituents are ignored) is approximately 0.02 electron volts which is in agreement with the finding that the average deviation between determinations for the same compound was approximately 0.03 electron volts.

The corresponding extrathermodynamic relationship is given by (see table 1):

$$pP_{I}=\frac{N_{0}\left(IP\right)}{2.303RT}=pP_{I}^{0}+\rho \sigma^{+}.$$ (14)

The results of correlating the data by eq (14) are given in table 3. In this case, two results are shown, one including all the data and one in which the amino substituent values are excluded. A test for outliers [59] (95% confidence interval) indicates that the amino substituted compounds come from a population different from the others. This lack of agreement for the amino substituents has been found in correlating the effect that aryl substituents have on the electron spin resonance spectra of *N*,*N*-dimethylanilinium radical cation [36], a topic that will be discussed below.

### Polarography of Aromatic Amines

An indirect method for assessing the relative ionization potentials of aromatic amines involves polarography. According to Foster [60] “polarographic oxidations may provide, by their experimentally determined half-wave oxidation potentials $E_{1/2}^{ox}$, a measure of the ionization potential of a compound.” If certain requirements are met, then the following equation holds:

$$E_{1/2}^{0x}=IP+\Delta F_{\text{solv}}+\text{constant},$$ (15)

where “Δ*F*_solv_ is the difference in solvation-energy between the compound and its positive ion. If it is assumed that, for a series of compounds, variations in *IP* are much greater than Δ*F*_solv_, then $E_{1/2}^{ox}$ may be used as a measure of the electron-donating ability of a donor. Because of this restriction it is obviously advisable to compare compounds which are chemically related” [60]. Correlation of the polarographic halfwave potentials of a series of aryl singly and multiply substituted *N*,*N*-dimethylanilines in acetonitrile [61] with ∑*σ*^+^ of the substituents gives results shown in table 3. In this case, the *p*-dimethylamino group gives a result consistent with other substituents. In contrast to this, Lagutskaya and Dadali [62] discuss their own and previous efforts to correlate the half-wave potentials of aromatic amines dissolved in aqueous solutions of various pH with *σ* and *σ*^−^ values of the substituents and remark that a poor correlation is obtained. No mention of *σ*^+^ values was made. According to Zuman [63], who has probably succeeded more than anyone else in correlating polarographic data, in describing the reactivity of benzene derivatives, the use of *σ*^+^ is needed “in those so far rare examples in which the reaction constant *ρ* is negative,” which in Zuman’s terminology indicates polarographic oxidation. (Zuman employs a sign convention opposite the one in this paper.)

### Electron Spin Resonance Spectroscopy

A practical way to evaluate the effects of substituents on the properties of free radicals is through the use of electron spin resonance (ESR) spectroscopy [36]. From observed coupling constants, a measure of the electronic spin density of the atom possessing an odd electron is ascertainable. Janzen correlated the nitrogen ESR hyperfine coupling constants of *meta*- and *para*-substituted *N,N*-dimethylanilinium cation radical against *σ^+^* and obtained a good correlation if the amino groups were excluded [36]. The only substituents to be at a considerable distance from the regression line were *m*-OH, *m*-CO_2_H and *m*-CO_2_C_2_H_5_. He also obtained ESR data for substituted nitrobenzene anion radicals to be discussed in section 6.

### Ortho Substitution

In the case of *ortho* substitution both electron- donating ability and a steric effect of the substituent may play a role, as discussed in section 4. This steric effect causes the reacting site, in this case, the amino group, to be out of the plane of the benzene ring [64]. A similar effect occurs when a dialkylamino group acts as a substituent [65]. Since the steric effect depends on the sizes of the aryl and the nitrogen substituents, it would be difficult, if not impossible, to correlate such data with respect to substituent constants and it will not be attempted in this investigation.

A simple rule to predict if steric interference preventing the coplanarity of the aromatic ring and the nitrogen substituents is absent is an adaptation of “the rule of six” [66]. In a secondary or tertiary aromatic amine, consider each of the atoms attached to the benzene ring at the *ortho*-position as number 1. A count of the attached atoms in succession shows that the atoms attached to the *α*-carbon of each of the nitrogen substituents are number 6. If the number 1 atoms and two of the number 6 atoms in each substituent are hydrogen (or are absent as in the case of the secondary amine), molecular scale models indicate that steric interference is absent, no matter the rest of the molecule. Otherwise, steric interference may be present.

### Spectroscopic Evaluation

The only previous investigation in which spectroscopic data of EDA complexes of aryl-substituted anilines with another benzene derivative were correlated against *σ*^+^ of the substituent is that of Kravtsov and Faingor [14], who correlated the frequencies of the absorption maxima of complexes of *para*-substituted *N*,*N*-dimethylanilines with trinitrobenzene in chloroform versus *σ*^+^ of the *para*-substituents. The extrathermodynamic relationship for their data is given in table 3.

Finally, it should be noted that Farrell and Newton proposed that the charge-transfer transition energies of the aniline complexes of tetracyanoethylene in chloroform be used to assess the ionization potentials of the amines [67]. However, the slope obtained from their data for *para*-substituted *N*,*N*-dimethylanilines (summarized in table 3) is inconsistent with that from the data of another investigation [14] and with the slopes from the extrathermodynamic relationships correlating ionization potential data [58]. The reason may be as follows. In the case of complexes of aromatic amines with other aromatic compounds, the amine is assumed to act as an *n*-donor with the electrons primarily of the nitrogen atom involved in the complexation. On the other hand, it has been shown that tetracyanoethylene reacts with aromatic amines at the *para*-position by, according to one hypothesis, first forming a *π*-complex, then a *σ*-complex and finally a new compound [68]. Thus the ionization potentials calculated from the transition energies of these latter complexes are not readily related to those obtained by other means.

## 6. Electron Affinities of Electron Acceptors and Extrathermodynamic Relationships

### Previously Reported Correlations

In comparison with studies on electron-donating aromatic amines, no exact relationship has apparently been published that correlates the *c-t* transition energies of complexes with the substituents of the electron acceptor if the acceptor is a benzene derivative.

However, approximate techniques have been derived. Lepley and Thelman [33] noted that the electron affinity of an electron acceptor generally increases with the number of strong electron withdrawing substituents and the location of these substituents in the acceptor structure. Based on the previous investigation of Hammond [69], who correlated the frequencies of the charge-transfer absorption peaks of complexes of mono-substituted *p*-benzophenones with hexamethylbenzene with *σ_p_* of the benzophenone substituent, Lepley and Thelman postulated that the electron affinity of a benzene derivative could be related to *σ_p_* of the substituent but they did not report any mathematical relationship. Argentar and Bowen used the sum of the values of the substituents of aromatic compounds functioning as electron acceptors, the geometry being ignored, in correlating the *c-t* transition energies of complexes of *N*,*N*-dimethyl-*p*-toluidine by a linear regression [26]. In these cases, the electron acceptors were assumed to function as *π*-acceptors.

According to Kosower (mentioned above in section 4) the *c-t* transition energies of 1-alkyl, 3- and 4-substituted pyridinium iodides, which form a charge-transfer complex by themselves, can be correlated with the *σ*^−^ values of the ring-substituents by assuming that the 3- and 4-positions of the pyridinium ring are analogous to the *meta*- and *para*-positions of an aromatic compound, respectively [45–47].

### Determination of the Exact Extrathermodynamic Relationship

Before attempting to derive the extrathermodynamic relationship for the substituent effect on the electron acceptor, the correct set of “sigma” values for the substituent must be established. Since there is no precedent for this, the correlation of ESR data as previously stated in the case of aromatic amines (sec. 4) furnishes this information indirectly. Janzen [36] showed that for *meta*- and *para*-substituted nitrobenzene anion radicals the nitrogen hyperfine coupling constants form two lines when correlated with *σ*^−^ of the substituent. Most of the values fall on one line. However, the unsubstituted compound and the two dinitro compounds form a second line, which intersects the first at the value of the unsubstituted nitrobenzene. This was explained by assuming that the second nitro group has more than just a minor perturbing influence on the nitro group that has gained an electron. The use of *σ*^−^ has also been reported by others in correlating the coupling constants of nitrobenzene anion radicals [70 and references therein]. The assumption that the logarithms of the coupling constants are correlated with the substituent constants [71] requires that new ad hoc substituent constants be defined and is therefore to be avoided.

### Polarography of Electron Acceptors

Analogous to the situation regarding aromatic amines discussed in (sec. 4), polarographic measurements provide a relative measure of the electron affinity. “By analogy with one-electron oxidation potentials … determinations of polarographic one- electron reduction potentials, $E_{1}^{\text{red}}$, whilst not directly measuring *E*, nevertheless provide one of the few independent experimental estimates of *E* which are available for a range of acceptor species [72].” In this quotation, *E* is the electron affinity. “Within experimental error, the reduction potential $\left(E_{1}^{\text{red}}\right)$ is equal to the half-wave potential $\left(E_{1/2}^{\text{red}}\right)$. For measurements against a standard calomel electrode:

$$E_{1/2}^{\text{red}}=E_{1}^{\text{red}}=E-\Delta F_{\text{solv}}-\varphi_{\text{Hg}}-E_{\text{Hg}:{\text{Hg}}^{++}}$$ (16)

where Δ*F*_solv_ is the difference in solvation energy between the compound and its negative ion, and represents mainly the solvation of the anion. *φ*_Hg_ is the work function [for the half-reaction:]^2^ *e*^−^ (in Hg)→ Hg_liq_ + *e*^−^ (equal to 4.54 eV) and $E_{\text{Hg}:{\text{Hg}}^{++}}$ is the absolute value of the calomel electrode (equal to 0.53 V). For two different acceptors *A*_1_ and *A*_2_, is Δ*F*_solv_ is assumed to be constant, then [72]”

$$EA\left(A_{1}\right)-EA\left(A_{2}\right)=E_{1}\left(A_{1}\right)-E_{1}\left(A_{2}\right)$$ (17)

In discussing substituent effects on the polargraphic reduction potentials of aromatic compound containing a reducible group having a double bond conjugated with the benzene ring, Zuman indicated that *σ*^−^ should be the preferable substituent constant for correlating the data [73]. However, in some instances, experimental evidence was available for preferring *σ* over *σ*^−^ [73]. In no instance was there mentioned an equation of the form shown as Equation (9′D) although linear structure-property equations were used for treating this type of data.

The electron affinities and polarographic half-wave potentials of several aryl substituted methyl benzoates have been measured [74]. However, there was no mention of correlating the data with the substituent parameters. These are correlated here and the results given in table 5. In this set of data and the following, the *σ*^−^ value of the *ortho*-substituent is set equal to *σ_p_*. In one previous investigation where steric factors were not important, *σ_o_* – *σ_p_* was approximately constant (as discussed in sec. 3).

Peover has determined the half-wave reduction potentials of a number of substituted nitrobenzenes [75]. The result of correlating the data by an extrathermodynamic relationship is given in table 5. Although *ortho*-substituents are apparently well-behaved in these instances, further investigation is needed to determine the limitations in assuming that $\sigma_{o}^{-}$ equals *σ_p_.*

### Spectroscopic Evaluation

In apparently the only investigation of its type, Peover obtained the *c-t* transition energies of complexes of substituted nitrobenzenes with *N*,*N*,*N*,*N*-tetramethylphenylenediamine and correlated the energies with the polarographic half-wave reduction potentials of the electron acceptor [75]. No correlation of the energies directly with substituent parameters was reported. The results of correlating the calculated *pE_T_* values versus *σ*^−^ of the *meta*- or *para*-substituent is given in table 5.

## 7. Effect of Solvent on the *C*-*T* Transition Energy — Solvatochromic Relationships

The most popular equation for correlating the effect of solvent on the transition energy of charge-transfer complexes is that due to McRae [76] as reviewed by Mataga and Kubota [77]. This is given as:

$$\begin{array}{l} \Delta v_{\text{abs}}=v_{\text{abs}}-v_{\text{abs}}^{g}\\ =\left(A+B+C\right)\left[\frac{n^{2}-1}{2n^{2}+1}\right]+E_{\text{abs}}\left[\frac{D-1}{D+2}-\frac{n^{2}-1}{n^{2}+2}\right]\\ +F_{\text{abs}}{\left[\frac{D-1}{D+2}-\frac{n^{2}-1}{n^{2}+2}\right]}^{2} \end{array}$$ (18)

where Δ*v*_abs_ is the difference in the frequency at which maximum absorption occurs in solution relative to that of the complex in vacuum (gas phase); *A*, *B*, *C*, *E*_abs_, and *F*_abs_ are constants dependent only on the solute; *n* and *D* are the refractive index and dielectric constant of the solvent, respectively.

In general, the quadratic term is negligibly small [78] so that this equation is approximately:

$$v_{\text{abs}}=v_{\text{abs}}^{g}+C_{1}\left[\frac{n^{2}-1}{2n^{2}+1}\right]+C_{2}\left[\frac{D-1}{D+2}-\frac{n^{2}-1}{n^{2}+2}\right]$$ (18′)

This equation has also been expressed as:

$$\Delta hv_{ct}=\left(AL_{0}+B\right)\left[\frac{n^{2}-1}{2n^{2}+1}\right]+C\left[\frac{D-1}{D+2}-\frac{n^{2}-1}{n^{2}+2}\right].$$ (19)

In this form Aihara et al. [78] correlated the solvent effects of the naphthalene-tetracyanoethylene and pyrene-tetracyanoethylene complexes, obtaining two straight lines, with hydrocarbons and halogenated hydrocarbons forming one series and carbonyl-containing compounds forming a second.

Emslie and Foster [8] correlated the frequencies of the ultraviolet absorption peaks of four complexes, of which two were of the aniline-substituted benzene type, against Kosower’s *Z* value [79] of the solvent. This value is the transition energy (in kcal/mol) of the charge-transfer complex, 1-ethyl-4-carbomethoxy-pyridinium iodide, dissolved in the solvent. They obtained two sets of straight lines depending on whether the solvents contained hydroxyl groups. Some nucleophilic solvents, e.g., acetone, did not fit the correlation.

Moriguchi et al. [80] correlated the solvent effects on the hexamethylbenzene-chloranil and hexamethyl-benzene-tetracyanoethylene complexes and obtained linear relationships between $v_{\max}^{ct}$ and *n*, (*n*^2^ − 1)/(2*n*^2^ + 1) or Kosower’s *Z* value. They also showed that an excellent correlation (correlation coefficient = 0.999) existed between (*n*^2^ − 1)/(2*n*^2^ + 1), (*n*^2^ − 1)/(*n*^2^ + 2) and *n* for the common solvents tested.

Koppel and Palm [81] have suggested the use of a multiparameter solvent effect relationship:

A=Ao+yY+pP+eE+bB (20)

where *A* is the measured property or a simple mathematical function thereof; *Y* is the polarity of the solvent, (*D −* 1)/(*D +* 2) or (*D −* 1)/(2*D* + 1); *P* is the polarizability, (*n^2^ −* 1)/*(n^2^* + 2); *E* is the electrophilic solvation power, which is based on Dimroth’s solvent polarity parameter, *E_T_* [82] and which is used in this case primarily to measure the ability of the solvent to hydrogen-bond to the solute; *B* is the nucleophilic solvating power (Lewis basicity) defined as $v_{\text{OD}}^{0}-v_{\text{OD}}$ where *v*_OD_ is the infrared stretching frequency of a hydrogen-bond donor group (e.g., the OD band of monomeric CH_3_OD) in the gas phase and *v*_OD_ is the corresponding value in the solvent; and *A*_0_, *Y*, *p*, *e* and *b* are empirical constants obtained by least-squares fitting the equation. For solvatochromic shifts in electronic spectra, the functions (*D* − 1)/*(D* + 2) and (*n*^2^ − 1)/(*n*^2^ + 2) were preferred.

In correlating the transition energies of *N*-phenyl pyridinium betaine (*E_T_* parameters) and the chargetransfer bands of 1-ethyl-4-carbomethoxypyridinium iodide complexes (*Z* parameter), the values of *y*, *p*, and *e* are not substantially different for the two sets of data [81]. Interestingly, statistical analysis [54] infers that in both cases *p* is not significantly different from zero at the 95 percent confidence level, indicating that for these systems, the refractive index is unimportant.

In order to obtain the relevant solvatochromic relationship, the *pE_T_* values calculated from the spectroscopic data for the complexes of *N*,*N*-dimethylaniline with 1,3,5-trinitrobenzene or 1,2,4,5-tetracyanobenzene studied by Emslie and Foster [8] were correlated using a simplified form of Koppel’s equation in which the *B* term was eliminated. This deletion was made because there are few solvents for which this value is known. Furthermore, this term was found necessary in very few previous cases [81]. Values of *n*, *D*, and *E* with the following exceptions were taken from the compilation of Koppel [81]; the refractive index of chloroform was taken as 1.4459 [83] and the values of *E* for chloroform, 1,1-dichloroethane and 1,1,2,2-tetrachloroethane were taken as 3.04, 3 and 3, respectively. The results are shown in table 4.

Statistical analysis [54] implies (at the 95 percent confidence level) that only the coefficient for the refractive index term is significant different from zero in the case in which 1,3,5-trinitrobenzene is the electron acceptor and that the coefficients for both refractive index and dielectric constant terms are significantly different from zero in the second case. Further analysis [84] indicates (at the 95 percent confidence level) that the intercepts and the corresponding slopes in the two cases are not significantly different from each other.

Since the standard errors of regression in the two cases are not significantly different from each other (at the 95 percent confidence level) [85] the data for the two complexes may be pooled and fitted by the equation

$$\begin{array}{l} pE_{T}=pE_{T}^{0}+\Delta pE_{T}X+y\left[\frac{D-1}{D+2}\right]\\ +\Delta yX\left[\frac{D-1}{D+2}\right]+p\left[\frac{n^{2}-1}{n^{2}+2}\right] \end{array}$$ (20)

where $pE_{T}^{0}$ is the intercept, which estimates the extrathermodynamic function of the *c-t* transition energy of the trinitrobenzene complex in a vacuum; *X* is an indicator variable set equal to *zero* in the case of the trinitrobenzene complex and to *one* otherwise, and $\Delta pE_{T}^{0}$ and Δ*y*, which are obtained by least-squares fitting of the equation, are the differences in the $pE_{T}^{0}$ and *y* values for the trinitrobenzene and tetracyanobenzene complexes respectively. These results are given in table 6. Statistical analysis [53] implies that (at the 95 percent confidence level) *y* and Δ*y* are not significantly different from zero. Repeating the regression after eliminating the Δ*y* term gives the final result shown in table 4. This correlation indicates that the effect of solvent on the *c-t* transition energies of the two complexes is parallel.

A correlation in which the *y* term was eliminated (not shown) indicates that the slopes of the dielectric constant terms are significantly different for the two complexes, and that the slope of the dielectric constant term for the trinitrobenzene complex is zero. Although this latter correlation is consistent with the data, the closeness of the slopes of the refractive index term in the two cases tends to persuade one that the slopes of the dielectric constant term should also be similar. More data on the transition energies of other complexes in this series in various solvents is required before it can be decided definitely that the solvent effects on this type of complex are indeed the same in all cases, i.e., that *y* and *p* always have the same values (within experimental error) as those found in this investigation.

These results along with those of Koppel [81] indicate that in correlating the charge-transfer transition energies of complexes between species that are uncharged in the ground state, the refractive index term is of primary importance; with species that are charged, the dielectric constant term is of supreme importance.

## 8. Summary and Conclusions

The mathematical relationships for the variations in the charge-transfer transition energy of a complex between an aromatic amine and a benzene derivative possessing an electron-accepting group may be summarized as follows:

For changes only in

1. Nitrogen substituent of the amine
  $$pE_{T}=pE_{T}^{0}+\rho \Sigma \sigma *$$ (22)
2. Aryl substituent of the amine
  $$pE_{T}=pE_{T}^{0}+\rho \Sigma \sigma^{+}$$ (23)
3. Substituent of the electron-accepting benzene derivative (with the electron-accepting group kept constant)
  $$pE_{T}=pE_{T}^{0}+\rho \Sigma \sigma^{-}$$ (24)
4. Solvent
  $$pE_{T}=pE_{T}^{0}+a\left[\frac{n^{2}-1}{n^{2}+2}\right]+b\left[\frac{D-1}{D+2}\right].$$ (25)

In these four equations, *pE_T_* is the extrathermodynamic function of the charge-transfer transition energy defined as *E_ct_*/2.303*RT*, where *E_ct_* is the transition energy (equal to $N_{0}hv_{\max}^{ct}$ where *N*_0_ is Avogadro’s number, *h* is Planck’s constant, and $v_{\max}^{ct}$ is the frequency in the ultraviolet or visible spectrum at which the differential absorption of the complex is maximum), *R* is the ideal gas constant and *T* is the temperature (K); σ^*^, *σ*^+^, and *σ*^−^ are the appropriate substituent parameters available in the literature; *n* and *D* are the refractive index and dielectric constant of the solvent, respectively, and $pE_{T}^{0}$, *ρ*, *a* and *b* are parameters obtained by least-squares fitting the line. For this system, *a* is much greater than *b.*

For changes, either in the nitrogen substituent of the amine or in the aryl substituent of either compound, the implicit assumption is that the substituent does not prevent coplanarity of the reacting group and the benzene ring. In addition, anomalous results may possibly occur if: for the amine, the $\sigma_{p}^{+}$ value of the aryl substituent is approximately equal to, or less than, the $\sigma_{p}^{+}$ value of the substituted amino group, which acts as the electron source; and for the electron acceptor, the $\sigma_{p}^{-}$ value of the substituent is approximately equal to, or greater than, the $\sigma_{p}^{-}$ value of the electron-accepting group of the parent compound.

The extrathermodynamic relationships for the electron-donating ability of the amine in the chargetransfer process with respect to variation in the aryl substituent is

$$pE_{D}=pE_{D}^{0}+\rho \Sigma \sigma^{+}$$ (26)

where *pE_D_* is the extrathermodynamic function of the ionization potential, *IP*, or of the polarographic oxidation half-wave potential, $E_{1/2}^{\text{ox}}$, of the electron donor equal to *N*_0_(*IP*)/2.303*RT* or $FE_{1/2}^{\text{ox}}/2.303RT$, respectively.

The corresponding relationship for the electron- accepting ability of the electron acceptor with respect to variations in the nonreacting substituent is

$$pE_{A}=pE_{A}^{0}+\rho \Sigma \sigma^{-}$$ (27)

where *pE_A_* is the extrathermodynamic function of the electron affinity, *EA*, or of the polarographic reduction half-wave potential, $E_{1/2}^{\text{red}}$, of the electron acceptor equal to *N*_0_(*EA*)/2.303*RT* or $FE_{1/2}^{\text{red}}/2.303RT$, respectively.

The same requirements of coplanarity and of the restrictions on the magnitudes of the sigma values of the substituent mentioned for the *c-t* transition energy of a complex hold in the above cases also.

Note added in proof: After the present investigation was completed, Davidson [86] proposed that the mechanism illustrated in figure 2 was the first step in the photoreaction of aromatic nitrocompounds with *N*-alkylanilines to yield primary aromatic amines.

## Figures and Tables

**Table 1:** Measured physiochemical properties of electron donors, acceptors and donor acceptor complexes and their associated energy an extrathermodynamic (*ET*) function of their energy

Material investigated	Property measured	Symbol	Associated energy, *E* (J/mol^−^)^a^	*ET* function symbol^b^
				
Electron donor	Ionization potential	*IP*	*N*_0_(*IP*)	*pP_I_*
	Polarographic half-wave potential, oxidation	$E_{1/2}^{\text{ox}}$	$FE_{1/2}^{\text{ox}}$	c*pE*
Electron acceptor	Electron affinity	*EA*	*N*_0_(*EA*)	*pA_E_*
	Polarographic half-wave potential, reduction	$E_{1/2}^{\text{red}}$	$FE_{1/2}^{\text{red}}$	e*pE*
EDA complex	Frequency at maximum absorption in the ultraviolet or visible spectrum	$v_{\max}^{ct}$	d $N_{0}hv_{\max}^{ct}$	*pE_T_*
	Wavelength at maximum absorption in the ultraviolet or visible spectrum	$v_{\max}^{ct}$	d $N_{0}hc/\lambda_{\max}^{ct}$	*pE_T_*

a Meaning of symbols: *N*_0_, Avogardro’s number (6.023 × 10^23^ molecules/mol); *h*, Planck’s constant (6.626 × 10^−34^ Js); *c*, the speed of light in a vacuum (2.998 × 10^8^ m/s): *F*, Faraday’s constant (9.649 × 10^4^ J/V).

b The *ET* function equals *E*/2.303*RT*, where *E* is the energy (or free energy) associated with a given process; *R* is the gas constant [8.314 J/(mol − K)]; *T* is the temperature (K).

c See ref. [48].

d See ref. [29].

e All values of physical constants taken from “ASTM Metric Practice Guide”, NBS Handbook 102 (1967).

**Table 2:** Characteristics of amine systems correlated in this investigation

Regression number	Parent amine^a^	Substituents	Electron acceptor	Solvent	Temperature	Ref.
						
1.	*N*,N-Dimethylaniline	*N*-CH_3_; *N*,*N*-(CH_3_)_2_; *N*,*N*-(C_2_H_5_)_2_; *N*,*N*-(*n*-C_3_H_7_)_2_; *N*,*N*-(*n*-C_4_H_9_)_2_	2,4,6-trinitrotoluene	cyclohexane	18–20° b	[1]
2.	*N*,*N*-Dimethylaniline	H; *N*-CH_3_; *N*-C_2_H_5_; *N*-(*n*-C_3_H_7_); *N*-*(iso*-C_3_H_7_); *N*-(*n*-C_4_H_9_); *N*,*N*-(CH_3_)_2_; *N*,*N*-(C_2_H_5_)_2_; *N*,*N*-(*n*-C_3_H_7_)_2_; *N,N-(n*-C_4_H_9_)_2_	1,3,5-trinitrobenzene	……do………………	……do………………	[1]
3. a.	*N*,*N*-Dimethylaniline	H; *p*-CH_3_; *p*-NHCOCH_3_; *p*-NH_2_; *p*-I: p-Br; *p*-Cl; *p*-OCH_3_	trinitrobenzene	chloroform	not reported^d^	[14]
b.	……do……………………	(c)	……do……………………	……do………………	……do………………	[14]
4. a.	Aniline	H; *m*-NO_2_; *p*-N0_2_; *m*-CH_3_; *p*-CH_3_ *m*-NH_2_, *p*-NH_2_; *p*-OCH_3_	………………………	vacuum	not reported^e^	[58]
b.	……do……………………	(c)	………………………	……do……………………	……do……………………	[58]
5. a.	*N*,*N*-Dimethylaniline	H; *m*-OCH_3_; *p*-OCH_3_; 3,4-(OCH_3_)_2_; 3,5-(OCH_3_)_2_; *p*-*N*(CH_3_)_2_	………………………	acetonitrile (Ag°/0.01M Ag^+^)	room temperature	[61]
b.	……do……………………	(c)	……………………….	……do……………………	……do……………………	[61]
6.	*N*,*N*-Dimethylaniline	H; *p*-OCH_3_; *p*-CH_3_; *p*-NO_2_; *p*-Cl	……………………….	acetonitrile (satd. calomel electrode)	not reported^f^	
7.	*N*,*N*-Dimethylaniline	H; *m*-CH_3_; 3,5-(CH_3_)_2_	tetracyanoethylene	chloroform	24°	[67]
8.	*N*,*N*-Dimethylaniline	H: *p*-CH_3_; *p*-C_2_H_5_; *p-iso*-C_3_H_7_; *p-t*-C_4_H_9_; *p*-F; *p*-Cl; *p*-Br; *p*-I	……do……………………	……do……………………	……do……………………	[67]

a The parent compound is that compound in the family taken as the standard; the intercept in the regression results shown in table 3 is an estimate of the extrathermodynamic function of the energy under consideration involving this compound.

b Since most of the values were obtained at 20°, this temperature was used.

c Same as above except NH_2_ or N(CH_3_)_2_ substituent(s) omitted.

d In all cases, unless otherwise indicated, when no temperature is reported, a temperature of 25° was used.

e In literature available from the manufacturer, 250° was recommended as the operating temperature. This temperature was used.

f Seo, E. T., Nelson, R. F., Fritsch, J. M., Marcoux, L. S., Leedy, D. W., Adams, R. N., J. Am. Chem. Soc. 88, 3498 (1966).

**Table 3:** Extrathermodynamic relationships of electron-donating aromatic amines

Regression number	*ET* function^a^	Substituent location	Substituent parameter or indicator variable	Slope^b^	Intercept^b^	S^c^	N^d^
							
1.	*pE_T_*	Nitrogen	σ*	6.9 ±2.3	48.53 ±0.57	1.24	5
2.a	*pE_T_*	Nitrogen	σ*	12.5 ±5.6	42.3 ±5.4	0.32	10
			e*x*_1_	1.7 ±4.5			
			f*x*_2_	2.7 ±4.6			
			g*n*_1_	−0.36 ±0.61			
2.b	*pE_T_*	Nitrogen	σ*	9.81 ±0.30	44.55 ±0.13	.36	10
3.a	*pE_T_*	Aryl	*σ^+^*	3.99 ±0.26	42.64 ±0.15	.36	8
3.b	*pE_T_*	Aryl	*σ^+^*	3.78 ±0.40	42.63 ±0.16	.37	7
4.a	*pP_I_*	Aryl	*σ*^+^	6.04 ±0.60	80.04 ±0.39	1.09	8
4.b	*pP_I_*	Aryl	*σ*^+^	6.41 ±0.12	80.273 ±0.067	0.16	6
5.a	*pE*	Aryl	*σ*^+^	5.70 ±0.65	8.41 ±0.53	1.02	6
5.b	*pE*	Aryl	*σ*^+^	5.0 ±1.2	8.29 ±0.59	1.09	5
6	*pE*	Aryl	*σ^+^*	7.56 ±0.88	13.40 ±0.46	1.02	5
7	*pE_T_*	Aryl	*σ*^+^	20.174 ±0.074	31.1956 ± 0.0063	0.0069	3
8	*pE_T_*	Aryl	*σ*^+^	−1.3 ±1.6	30.21 ±0.34	.91	9

a Defined in table 1.

b Least-squares estimate of the coefficient ± its standard error.

c Standard error of regression.

d Number of measurements.

e Indicator variable set equal to *zero* in the case of primary amine; set equal to *one* otherwise.

f Indicator variable set equal to *one* in the case of tertiary amine; set equal to *zero* otherwise.

g Number of hydrogen atoms on the *α*-carbon atom of the nitrogen substituent.

**Table 4:** Characteristics of electron acceptor systems correlated in this investigation

Regression number	Parent electron acceptor^a^	Substituents	Electron donor	Solvent	Temperature	Ref.
						
1.	nitrobenzene	H; *p*-Cl; *m*-NO_2_; *o*-NO_2_; *p*-NO_2_; 3-NO_2_-4-F; 3-NO_2_-2,4-F_2_; 3,5-(NO_2_)_2_	………………………………	acetonitrile (satd. calomel electrode)	25°	[75]
2.	do.	H; *p*-Cl; *m*-NO_2_; *p*-NO_2_; 3,5-(NO_2_)_2_	*N*,*N*,*N*,*N*-tetramethyl-*p*-phenylenediamine	methylene chloride	room temperature^b^	[75]
3.	methyl benzoate	*o*-CO_2_CH_3_; *m*-CO_2_CH_3_; *p*-CO_2_CH_3_; 2,3,5,6-Cl_4_-4-CO_2_CH_3_; *p*-CO_2_H	………………………………	dimethyl formamide (satd. calomel electrode)	not reported	[74]
4.	methyl benzoate	H; *o*-CO_2_CH_3_; *m*-CO_2_CH_3_; *p*-CO_2_CH_3_; 2,3,5,6-Cl_4_-4-CO_2_CH_3_	………………………………	gas phase	180–270°^c^	[74]

a The parent compound is that compound in the family taken as the standard; the intercept in the regression results shown in table 5 is an estimate of the value of the ET function of the energy under consideration involving this compound.

b In all cases, when the temperature was not reported, 25° was used in the calculation of the ET function.

c The average temperature of 193°C was used.

**Table 5:** Extrathermodynamic relationships for aryl-substituted nitrobenzenes and methyl benzoates

Regression number	*ET*^a^ function	Substituent location	Substituent parameter	Slope^a^	Intercept^a^	*S^a^*	*N*^a^
							
1.	*pE*	Aryl	*σ*^−^	−6.40 ±0.31	19.62±0.27	0.38	8
2.	*pE_T_*	Aryl	*σ*^−^	−12.10±0.35	50.93±0.35	.43	5
3.	*pE*	Aryl	*σ*^−^	5.9± 1.9	59.8±1.9	2.25	4
4.	*pA_E_*	Aryl	*σ*^−^	2.8± 1.1	4.0±1.0	1.58	5

a For explanation of terms, see table 2 footnotes.

**Table 6:** Solvatochromic relationships (*pE_T_* versus solvent properties) of electron donor-acceptor complexes of *N,N*-dimethylaniline with two benzene derivatives

Regression number	Electron acceptor^a^	Solvent parameter or indicator variable	Slope^b^	Intercept^b^	*S*^b^	*N*^b^
						
1.a	1,3,5-trinitrobenzene	*cY*	−0.65 ±0.55	56.1 ± 1.3	0.44	20
		d*P*	−43.5 ± 5.0			
		e*E*	0.016 ±0.032			
1.b	Same data as in 1.a	*Y*	−0.47 ±0.40	56.2 ± 1.2	0.44	20
		*P*	−44.1 ±4.8			
2.a	1,2,4,5-tetracyanobenzene	*Y*	−2.03 ±0.81	53.6 ± 1.8	0.65	20
		*P*	−42.9 ± 7.3			
		*E*	0.035 ± 0.047			
2.b	Same data as in 2.a	*Y*	−1.62 ±0.59	53.8 ± 1.8	0.64	20
		*P*	−44.1 ±7.0			
3.a	1,3,5-trinitrobenzene and	f*X*	−2.39 ±0.47	56.2 ± 1.1	0.54	40
	1,2,4,5-tetracyanobenzene	*Y*	−0.47 ±0.49			
		*XY*	−1.15 ±0.69			
		*P*	−44.1 ±4.2			
3.b	Same data as in 3.a	*X*	−3.13 ±0.18	56.6 ± 1.1	0.55	40
		*Y*	−1.04 ±0.36			
		*P*	−44.1 ±4.3			

a All raw data from ref. [8]. (The data for the solvent decanol was omitted because its *E* value is unknown.)

b For explanation of term, see Table 2 footnotes.

c The polarity of the solvent, estimated by (*D* − 1)/*(D* + 2), where *D* is the dielectric constant at 20 °C.

d The polarizability of the solvent, estimated by (*n*^2^ − 1)/(*n*^2^ + 2), where *n* is the refractive index at 20 °C.

e The electrophilic solvation power, which measures the ability of the solvent to hydrogen-bond to the solute.

f An indicator variable set equal to zero for the complex of 1,3,5-trinitrobenzene and to one, otherwise.

The use of these terms is explained in the text.

No temperature for the determination of the raw data was reported by the investigators. Since the solvent parameters, *Y* and *P*, were determined at 20 °C, *pE_T_* was calculated using this temperature.

## Footnotes

1 Figures in brackets indicate the literature references at the end of this paper.

2 Author’s editorial change.
